# Comparative Transcriptomics Identifies Novel Genes and Pathways Involved in Post-Traumatic Osteoarthritis Development and Progression

**DOI:** 10.3390/ijms19092657

**Published:** 2018-09-07

**Authors:** Aimy Sebastian, Jiun C. Chang, Melanie E. Mendez, Deepa K. Murugesh, Sarah Hatsell, Aris N. Economides, Blaine A. Christiansen, Gabriela G. Loots

**Affiliations:** 1 Physical and Life Sciences Directorate, Lawrence Livermore National Laboratories, Livermore, CA 95101, USA; sebastian4@llnl.gov (A.S.); JiunChiun.Chang@ucsf.edu (J.C.C.); mendez20@llnl.gov (M.E.M.); murugesh2@llnl.gov (D.K.M.); 2 School of Natural Sciences, UC Merced, Merced, CA 95101, USA; 3 Regeneron Pharmaceuticals, Tarrytown, NY 10020, USA; sarah.hatsell@regeneron.com (S.H.); aris.economides@regeneron.com (A.N.E.); 4 Department of Orthopedic Surgery, UC Davis Medical Center, Sacramento, CA 95101, USA; bchristiansen@ucdavis.edu

**Keywords:** osteoarthritis, RNA-seq, STR/ort, C57BL/6J, MRL/MpJ, ACL injury, PTOA, regeneration, inflammation, *B4galnt2*

## Abstract

Anterior cruciate ligament (ACL) injuries often result in post-traumatic osteoarthritis (PTOA). To better understand the molecular mechanisms behind PTOA development following ACL injury, we profiled ACL injury-induced transcriptional changes in knee joints of three mouse strains with varying susceptibility to OA: STR/ort (highly susceptible), C57BL/6J (moderately susceptible) and super-healer MRL/MpJ (not susceptible). Right knee joints of the mice were injured using a non-invasive tibial compression injury model and global gene expression was quantified before and at 1-day, 1-week, and 2-weeks post-injury using RNA-seq. Following injury, injured and uninjured joints of STR/ort and injured C57BL/6J joints displayed significant cartilage degeneration while MRL/MpJ had little cartilage damage. Gene expression analysis suggested that prolonged inflammation and elevated catabolic activity in STR/ort injured joints, compared to the other two strains may be responsible for the severe PTOA phenotype observed in this strain. MRL/MpJ had the lowest expression values for several inflammatory cytokines and catabolic enzymes activated in response to ACL injury. Furthermore, we identified several genes highly expressed in MRL/MpJ compared to the other two strains including *B4galnt2* and *Tpsab1* which may contribute to enhanced healing in the MRL/MpJ. Overall, this study has increased our knowledge of early molecular changes associated with PTOA development.

## 1. Introduction

Osteoarthritis (OA) is a painful degenerative joint disease that causes disability and diminishes the quality of life for millions of people worldwide [1]. Joint injury, particularly injuries to the anterior cruciate ligament (ACL), often result in post-traumatic osteoarthritis (PTOA) within 1–2 decades from the injury [2]. PTOA accounts for about 12% of all OA cases, yet the mechanisms contributing to PTOA after joint injury are not well understood, and there are currently no effective treatments available for PTOA [3]. Many people developing injury- or age-related OA do not show any symptoms until significant joint damage has occurred, and joint pain is not always indicative of OA [4]. For many diagnosed with OA, the only available treatment options are joint replacement surgery and/or pain management. Therefore, there is a dire need for the discovery of biomarkers that can facilitate early detection of the disease and new therapeutic strategies for the prevention of PTOA. While many factors can influence the development of OA, injury-mediated OA holds the greatest promise for the development of effective pharmacologic interventions because a treatment can be administered at the time of surgery or immediately post-injury.

Acute joint trauma triggers several molecular events over the course of the first 1–2 weeks post-injury, which directly or indirectly contribute to the subsequent cartilage damage that is characteristic of OA. An understanding of these early molecular events provides a basis for identifying potential biologic targets for intervention to prevent subsequent joint degeneration [5]. Characterization of gene expression changes during OA development and progression at the whole genome level will provide novel mechanistic insights that could be translated into disease-modifying therapies. Numerous studies have used human biopsy samples to gain new insights about joint OA pathogenesis [6,7,8]; however, there are limitations in terms of the types of studies that can be conducted using human subjects. Human samples are usually obtained during knee replacement surgery and therefore represent late stages of the disease. To overcome this gap in knowledge, animal models allow us to investigate OA development longitudinally and are particularly well suited to studying early molecular events to derive new insights into the key factors contributing to disease progression. Using a noninvasive tibial compression (TC) injury model [9] that closely mimics anterior cruciate ligament (ACL) rupture in humans, we recently profiled the genes expression in knee joints from C57BL/6J mice during the onset and progression of PTOA and identified the molecular changes that characterize early and late stages of PTOA [10], including enhanced inflammatory responses at early timepoints and cartilage and bone remodeling at both early and late timepoints. We also noted that a majority of the transcriptional changes happened within the first few weeks post-injury.

In this study, we used the TC injury model to study early molecular events associated with PTOA development in three mouse strains with varying susceptibility to OA: STR/ort (highly susceptible), C57BL/6J (moderately susceptible), and MRL/MpJ (not susceptible). The STR/ort mice develop OA spontaneously early in life and show many human OA characteristics, including proteoglycan loss, extracellular matrix (ECM) degradation, and subchondral sclerosis [11]. They also exhibit osteophyte formation, a phenotype more readily seen in animal models where the joint is not stabilized. The MRL/MpJ is a mouse strain with exceptional abilities to heal wounds made in multiple tissues without the production of a fibrotic scar [12]. MRL/MpJs are protected from PTOA and do not develop degenerative joint changes following articular fracture [13]. It has been suggested that MRL/MpJ mice possess an intrinsic ability to regenerate articular cartilage, yet the molecular mechanisms responsible for this phenotype have yet to be revealed [14]. To characterize genes that contribute to increased OA susceptibility in STR/ort and resistance to PTOA in MRL/MpJ and to understand the molecular changes associated with early stages of PTOA development in these mice, we profiled gene expression in the knee joints of MRL/MpJ, C57BL/6J, and STR/ort by RNA sequencing (RNA-seq) at 0-day, 1-day, 1-week and 2-weeks post-injury. Understanding the molecular and genetic basis of enhanced OA susceptibility in STR/ort and resistance to PTOA in MRL/MpJ will improve our understanding of PTOA pathogenesis and may highlight new treatment options for PTOA or identify biomarkers that track disease progression.

This study identified 944, 2330, and 2702 genes differentially regulated in MRL/MpJ, C57BL/6J, and STR/ort, respectively, in response to ACL injury, including 553 genes that were shared by all strains. We identified increased, persistent inflammation, elevated catabolic activity, and elevated apoptosis as significant contributors to PTOA development. This study also identified several genes that may contribute to enhanced healing and tissue regeneration, including *B4galnt2* and *Tpsab1*. Furthermore, this study identified several potential OA biomarkers, including *Mamdc2* and *Pxdn*.

## 2. Results

### 2.1. Evaluation of PTOA Development and Progression in STR/ort, C57BL/6J, and MRL/MpJ Mice Following ACL Injury

**Figure 1:**
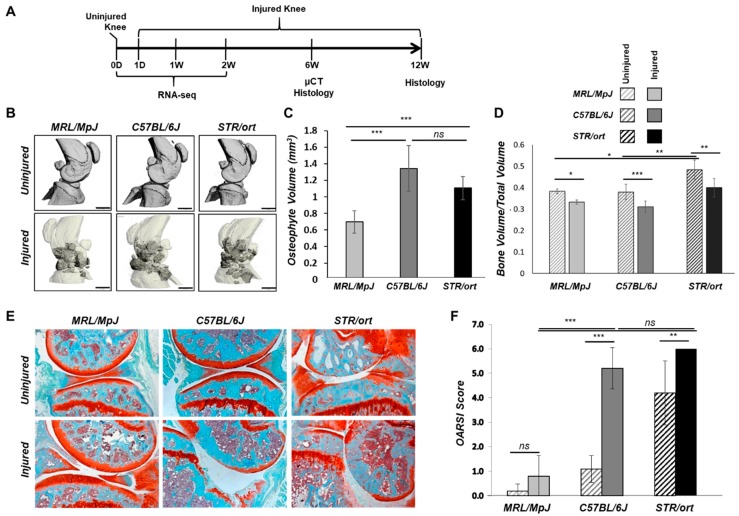
Anterior cruciate ligament (ACL) injury leads to post-traumatic osteoarthritis (PTOA) in C57BL/6J and STR/ort but not in MRL/MpJ mice. Knee joints were injured at 10-weeks of age. (**A**) Timeline for histology, µCT, and RNA-seq sample collection [0-day (0D), 1-day post-injury (1D), 1-week post-injury (1W), 2-weeks post-injury (2W), 6-weeks post-injury (6W), and 12-weeks post-injury (12W)]. (**B**) µCT representation of injured and uninjured joints at 6-weeks post-injury. Darker regions in the injured scans depict osteophytes. (**C**) Osteophyte volume quantification (dark regions in the injured scans in (**B**) at 6-weeks post-injury. (**D**) Epiphyseal trabecular bone volume ratio of the distal femur was quantified and analyzed between injured and uninjured joints at 6-weeks post-injury. (**E**) Histological assessment of uninjured and injured joints at 12-weeks post-injury using Safranin-O and Fast Green staining (5× magnification). (**F**) OARSI scoring of histological sections of injured and uninjured joints at 12-weeks post-injury. Scale bar = 1 mm. * *p* < 0.05, ** *p* < 0.01, *** *p* < 0.001, ns not significant.

Injured and uninjured contralateral joints of STR/ort, C57BL/6J, and MRL/MpJ mice were phenotyped using histology and/or microcomputed tomography (μCT) at 6- and 12-weeks post-injury to assess tissue morphology. Gene expression was profiled by RNA-seq before injury and 1-day, 1-week, and 2-weeks post-injury (Figure 1A). The joint damage was assessed by measuring the extent of osteophyte formation and the severity of cartilage degradation. Osteophyte formation was observed in all three strains by 6-weeks post-injury; MRL/MpJ injured joints had significantly less ectopic bone than the other strains (Figure 1B,C). In all three strains, injured joints lost significant subchondral bone volume in the femoral epiphysis relative to the uninjured contralateral joints (Figure 1D). Trabecular bone volume fraction (BV/TV) was significantly higher in STR/ort compared to the other two strains (Figure 1D), and STR/ort had a significantly higher bone mass than the other two strains, consistent with prior publications [15]. At 12-weeks post-injury, STR/ort contralateral joints displayed significant proteoglycan loss and cleft down below the superficial and into the mid zone of the tibial cartilage, whereas MRL/MpJ and C57BL/6J contralateral joints had healthy cartilage (Figure 1E). Injured joints of C57BL/6J and STR/ort exhibited severe cartilage erosion at 12-weeks post-injury (Figure 1E,F). In contrast, the MRL/MpJ displayed an insignificant proteoglycan loss, suggesting that MRL/MpJ are protected from ACL injury induced cartilage damage. MRL/MpJ injured joints were significantly different than C57BL/6J and STR/ort injured joints, but no statistical difference was found between C57BL/6J and STR/ort injured joints (Figure 1F).

### 2.2. Characterizing Genes Related to Osteoarthritis Susceptibility

**Figure 2:**
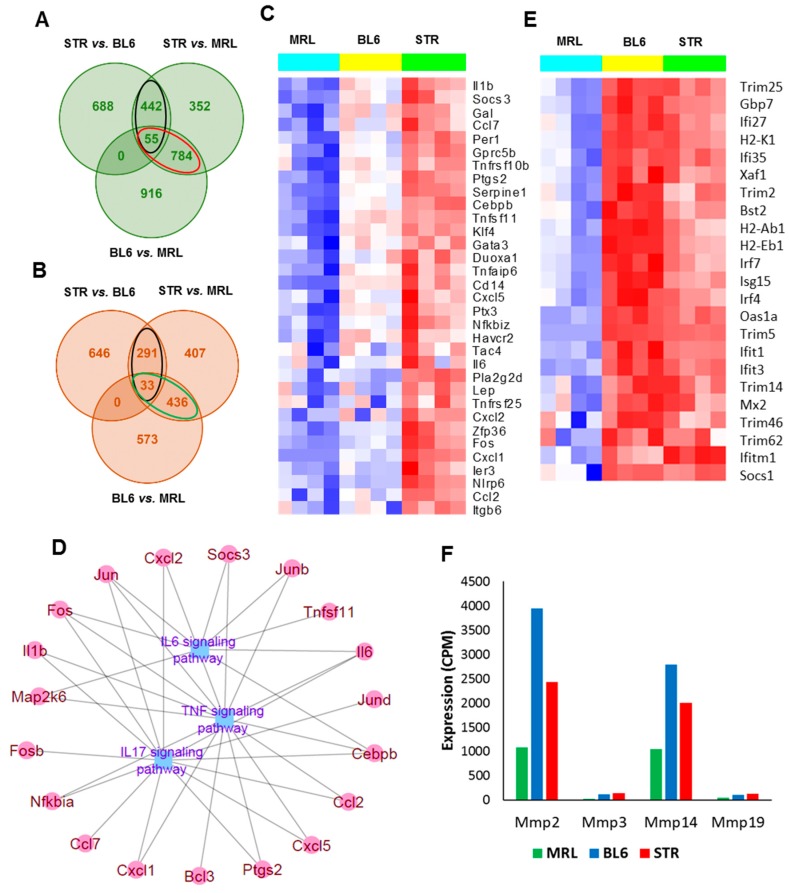
Genes associated with osteoarthritis susceptibility. (**A**) Overlap between genes upregulated in STR/ort (STR) compared to C57BL/6J (BL6) and MRL/MpJ (MRL) mice and in BL6 compared to MRL at 0-day. Genes upregulated in STR compared to both BL6 and MRL are shown in black ovals and genes downregulated in MRL (upregulated in both STR and BL6 compared to MRL) are shown in red ovals. (**B**) Overlap between genes downregulated STR compared to BL6 and MRL and in BL6 compared to MRL at 0-day. Genes downregulated in STR compared to both BL6 and MRL are shown in black ovals and genes upregulated in MRL (downregulated in both STR and BL6 compared to MRL) are shown in green ovals. (**C**) Inflammatory-response-related genes highly expressed in STR compared to BL6 and MRL at 0-day. (**D**) TNF, IL17, and IL6 signaling pathway-associated genes with high expression in STR compared to both BL6 and MRL. (**E**) Interferon signaling pathway-associated genes with low expression in MRL compared to both STR and BL6. (**F**) Matrix metalloproteinases (MMPs) with low expression in MRL compared to both STR and BL6.

To identify transcripts that correlate with OA risk, we profiled the knee joints of 10-weeks old uninjured MRL/MpJ, C57BL/6J and STR/ort mice and examined all pair-wise comparisons (Figure 2A,B, Table S1). Four hundred ninety-seven genes were up-regulated in STR/ort compared to both C57BL/6J and MRL/MpJ (Figure 2A). This included 33 ‘inflammatory response’ -related genes (*Il1b*, *Il6*, *Ccl2*, *Ccl7*, *Cxcl1*, *Cxcl2*, etc.) (Figure 2C) and 78 genes associated with ‘apoptotic process’ including *Egr1*, *Id3*, *Cebpb*, *Fos*, and *Jun* (Figure S1A). Genes up-regulated in STR/ort compared to the other two strains also showed enrichment for ontology terms ‘response to wounding’ (40 genes), ‘response to oxidative stress’ (24 genes including *Ptgs2*, *Rhob*, *Duox1*, *Areg* and *Plk3*), ‘response to hypoxia’ (19 genes), and ‘ossification’ (25 genes)’. Enriched signaling pathways associated with these genes include ‘TNF signaling pathway’ (15 genes), ‘IL17 signaling pathway’ (14 genes) and ‘IL6-mediated signaling events’ (8 genes) (Figure 2D). Genes down-regulated in STR/ort compared to the other two strains showed enrichment for biological processes such as ‘leukocyte activation’ (29 genes), ‘hemopoiesis’ (27 genes) and ‘immune response’ (39 genes including *Tlr1*, *Tlr5*, *Itgam*, *Itgad*, *Cd3d* and *Cd8a*).

MRL/MpJ had the lowest expression values for the majority of ‘inflammatory response’-related genes (Figure 2C). Genes with lowest expression in MRL/MpJ compared to other two strains (839 genes) also showed enrichment for ontology terms including ‘extracellular matrix organization’ (69 genes), ‘collagen catabolic process’ (18 genes), ‘response to wounding’ (71 genes), ‘immune response’ (98 genes), and ‘cytokine production’ (50 genes). Expression of interferon signaling pathway-associated genes, such as *Ifitm1*, *Ifit3*, *Ifi27*, *Ifi35*, *Trim2*, and *Trim5*, and matrix metalloproteinases (MMPs), including *Mmp2*, *Mmp3*, *Mmp14*, and *Mmp19*, were also significantly lower in MRL/MpJ compared to the other two strains (Figure 2E,F). Several genes involved in wound healing, including *Postn*, *Fn1*, *Dcn*, *Ctgf*, *Col3a1*, *Col5a1*, *Plat*, and *Hbegf* also exhibited lowest expression levels in MRL/MpJ (Figure S1B). Four hundred sixty-nine genes were upregulated in MRL/MpJ compared to both STR/ort and C57BL/6J (Figure 2B). This included 21 genes associated with ‘T cell activation’ (*Cd3d*, *Cd4*, *Jak3* etc.). Other enriched ontology terms associated with genes upregulated in MRL/MpJ included ‘lymphocyte aggregation’ (21 genes), ‘defense response’ (49 genes), ‘heme metabolic process’ (7 genes), and ‘cellular ion homeostasis’ (23 genes). Enriched pathways associated with the upregulated genes included ‘heme biosynthesis’ (4 genes) and ‘complement cascade’ (6 genes).

### 2.3. Early Molecular Changes Associated with PTOA Development in STR/ort, C57BL/6J, and MRL/MpJ Mice

**Figure 3:**
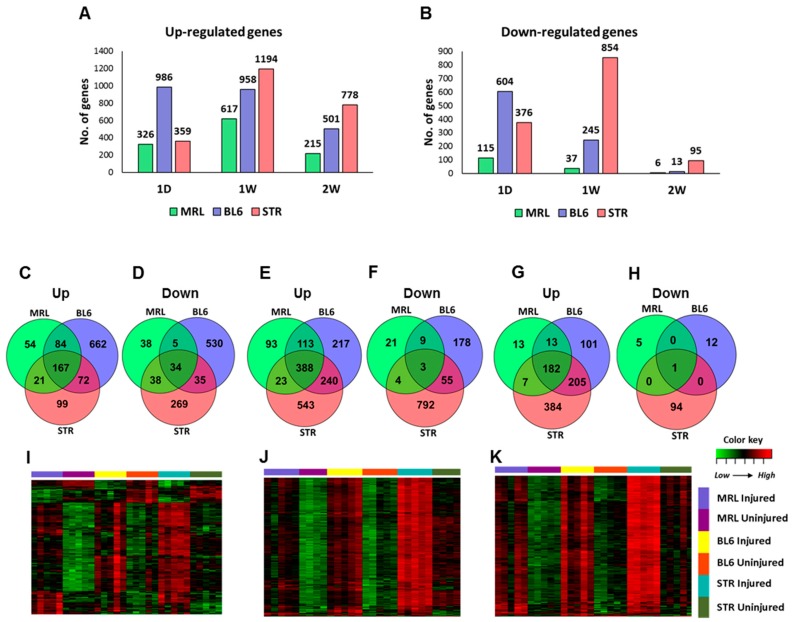
Early molecular changes associated with PTOA development. (**A**) Genes upregulated and (**B**) downregulated at 1-day (1D), 1-week (1W), and 2-weeks (2W) post-injury in STR/ort (STR), C57BL/6J (BL6), and MRL/MpJ (MRL). Overlap between genes (**C**) upregulated and (**D**) downregulated at 1-day post-injury. Overlap between genes (**E**) upregulated and (**F**) downregulated at 1-week post-injury. Overlap between genes (**G**) upregulated and (**H**) downregulated at 2-weeks post-injury. Genes upregulated in all three strains at (**I**) 1-day, (**J**) 1-week, and (**K**) 2-weeks post-injury. Majority of these injury-induced genes showed highest expression in injured STR.

**Figure 4:**
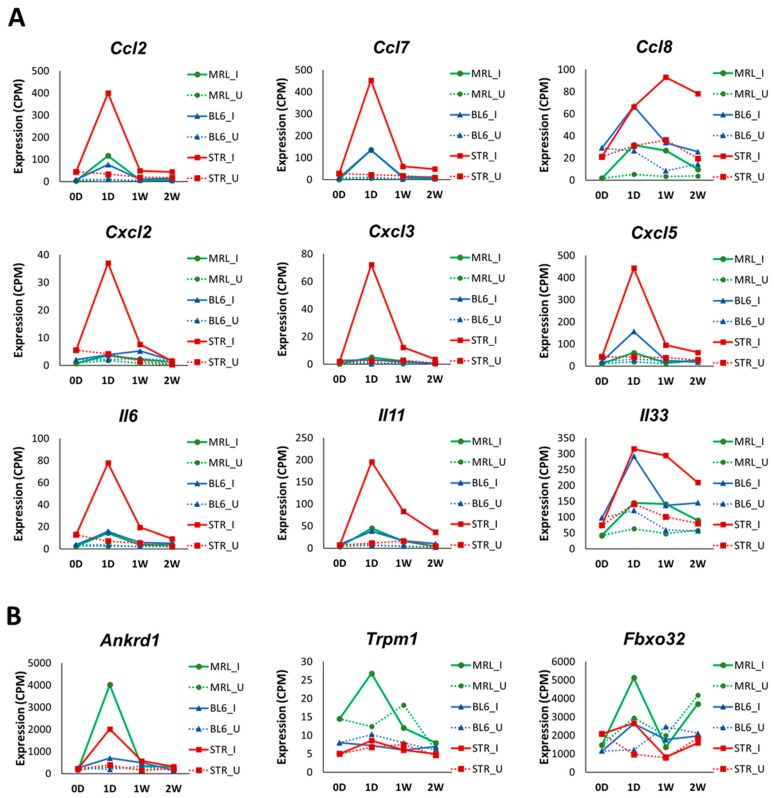
Expression profiles of inflammatory cytokines. (**A**) Selected inflammatory cytokines with significantly higher expression in injured STR/ort (STR) compared to C57BL/6J (BL6) and MRL/MpJ (MRL) at 1-day post-injury. (**B**) Selected injury-induced genes with significantly higher expression in injured MRL compared to the other two strains at 1-day post-injury. I: Injured; U: Uninjured; 0D: 0-day; 1D: 1-day; 1W: 1-week; 2W: 2-weeks.

To identify the early molecular changes associated with PTOA development in STR/ort, C57BL/6J, and MRL/MpJ mice, we examined gene expression changes in injured joints compared to uninjured contralateral joints at 1-day, 1-week, and 2-weeks post-injury. At 1-day post-injury, we identified 441 (326 up; 115 down), 1590 (986 up; 604 down), and 735 (359 up; 376 down) differentially expressed genes (>1.5 fold) in the injured joints of MRL/MpJ, C57BL/6J, and STR/ort, respectively, including 201 genes commonly changed in all three strains (Figure 3A–D, Table S2). Enriched biological processes associated with the upregulated genes included “extracellular matrix organization”, “vasculature development”, “cell migration”, “angiogenesis”, “response to wounding” and “inflammatory response” for all three strains and “granulocyte migration”, “reactive oxygen species metabolic process”, “cytokine secretion” and “response to tumor necrosis factor” for STR/ort alone. At 1-day post-injury, 59, 78, and 45 “inflammatory response”-related genes were upregulated in STR/ort, C57BL/6J, and MRL/MpJ, respectively. Several inflammatory cytokines, including *Ccl2*, *Ccl7*, *Ccl8*, *Cxcl5*, *Il6*, and *Il33*, were upregulated in all three genotypes in response to injury (Table 1). The majority of these transcripts showed significantly higher expression levels in injured STR/ort compared to the other two strains (Figure 4A). Furthermore, several immune/inflammatory response genes upregulated at 1-day remained elevated in STR/ort at later time points compared to uninjured controls; however, their expression returned to uninjured control level in C57BL/6J and MRL/MpJ strains by 1–2 weeks post-injury (Table 1). Only few injury-induced genes, including *Ankrd1*, *Trpm1*, and *Fbxo32*, showed highest expression in MRL/MpJ compared to the other two strains (Figure 4B). Genes downregulated in STR/ort injured joints compared to uninjured controls showed enrichment for “biomineral tissue development”, “muscle structure development” and “glycosaminoglycan metabolic process”, whereas genes downregulated in MRL/MpJ injured joints compared to contralateral joints showed enrichment for “pyruvate metabolic process”, “positive regulation of fatty acid oxidation”, and “nucleotide phosphorylation”.

At 1-week post-injury, 654 (617 up; 37 down), 1203 (958 up; 245 down), and 2048 (1194 up; 854 down) genes were differentially regulated in injured joints of MRL/MpJ, C57BL/6J, and STR/ort, respectively, relative to respective uninjured controls (Figure 3A,B,E,F). This included 269 genes commonly changed in all three genotypes (Table S2). At 2-weeks post-injury, 873 (778 up; 95 down) and 514 (501 up; 13 down) genes were differentially regulated in injured joints of STR/ort and C57BL/6J, respectively, whereas only 221 (215 up; 6 down) genes were found to be differentially regulated in injured joints of MRL/MpJ, and 183 of these genes overlapped with genes differentially expressed in the other two strains (Figure 3A,B,G,H). In addition, most of the genes differentially expressed at 2-weeks were upregulated, with less than 11% of differentially expressed genes being significantly downregulated in any strain (Table S2).

**Figure 5:**
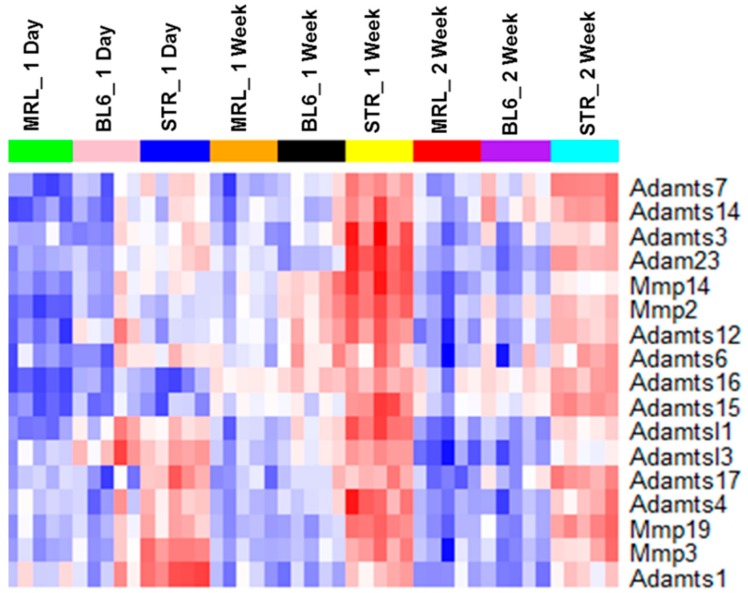
Matrix degrading enzymes showed highest expression in injured STR/ort. Expression of selected metalloproteinases in injured STR/ort (STR), C57BL/6J (BL6), and MRL/MpJ joints.

Although a large number of genes differentially expressed in response to injury were common to all three strains, STR/ort exhibited the highest expression values for a majority of these genes (Figure 3I–K). Several genes associated with “extracellular matrix organization”, “vasculature development”, “response to wounding”, “osteoblast differentiation”, “ossification”, and “collagen catabolic processes” were upregulated at 1-week and/or 2-weeks post-injury in all three strains. A number of catabolic enzymes, including *Mmp2*, *Mmp3*, *Mmp19*, *Adamts1*, and *Adamts4*, were upregulated in all three strains at 1-week and/or 2-weeks post-injury and the expression values of a majority of these catabolic enzymes were significantly higher in STR/ort compared to the other two strains (Table 2, Figure 5). At 1-week post-injury, genes associated with chondrocyte differentiation, including *Sox9* and *Runx1*, were upregulated and several muscle-related genes, including *Myh7*, *Myl2*, *Myl3*, *Myoc*, *Acta1*, and *Actc1*, were downregulated exclusively in STR/ort and C57BL/6J (Table S2). Several members of Wnt signaling, a major signaling pathway involved in skeletal development and bone metabolism, including Wnt receptor *Fzd2* and Wnt pathway inhibitors *Sfrp1* and *Sfrp2* were upregulated in all three strains at 1-week and 2-weeks post-injury (Table S2). It is likely that these genes play a significant role in cartilage and bone remodeling following ACL injury.

### 2.4. Potential Candidate Genes Associated with Enhanced Healing and Articular Cartilage Regeneration in MRL/MpJ

**Figure 6:**
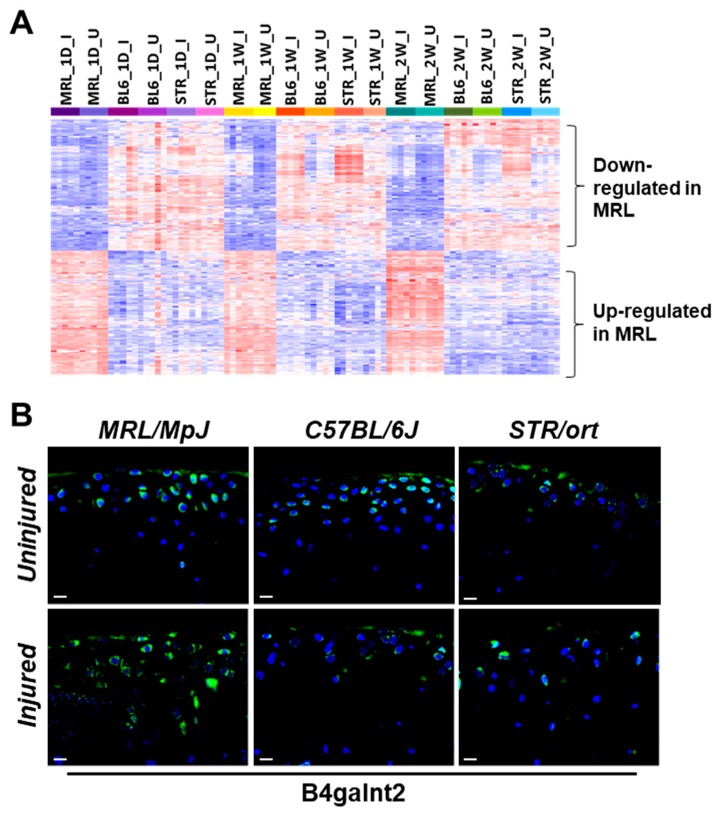
Candidates genes associated with enhanced healing in MRL/MpJ. (**A**) Genes differentially expressed in injured and uninjured MRL/MpJ (MRL) knee joints compared to both STR/ort (STR) and C57BL/6J (BL6) knee joints at all timepoints examined. (**B**) Elevated IHC expression of B4galnt2 in injured and uninjured joints of MRLs. Scalebar = 10 µm.

Compared to STR/ort and C57BL/6J, 204 genes were upregulated and 217 were downregulated in both injured and uninjured joints of MRL/MpJ at all timepoints examined (Table S3). ACL injury had little effect on the expression of majority of these genes (Figure 6A). Using microarrays, Cheng et al. profiled genes involved in digit amputation response in MRL/MpJ and C57BL/6 at 0-day (pre-amputation), 3-days, 1-week, and 2-weeks post-amputation and identified genes differentially expressed in digits at various timepoints postamputation compared to 0-day in both strains as well as genes differentially expressed between strains [16]. To characterize the genes that may contribute to enhanced healing and/or regeneration in MRL/MpJ, we examined the overlap between genes differentially expressed in MRL/MpJ compared to the other strains in both knee joints and digits at 0-day, 1-week, and 2-weeks post-injury. Our analysis identified five genes upregulated in MRL/MpJ, including *B4galnt2*, *Tpsab1*, *Vwa5a*, and *Aox4*, and 16 genes downregulated in MRL/MpJ, including *Mamdc2*, *Capg*, *Myoc*, and *Trim12a*, compared to the other strains in both datasets at all timepoints examined (Figure S2, Table 3). We further experimentally validated the differential expression of *B4galnt2*, a gene associated with muscle regeneration [17], in knee joints of all three strains. Immunohistochemistry confirmed that B4galnt2 (Figure 6B) was highly expressed in both injured and uninjured joints of MRL/MpJ compared to other two strains.

## 3. Discussion

STR/ort, C57BL/6J, and MRL/MpJ respond differently to knee joint injury. Here, we have introduced the first report that directly compares molecular and histological outcomes to a noninvasive ACL injury induced joint damage in these three strains. All three strains had deficits in epiphyseal trabecular bone in the injured joints and exhibited considerable osteophyte formation. STR/ort mice had degeneration in the contralateral joint and severe degeneration in the injured joint, whereas C57BL/6J mice had severe degeneration in the injured joint but not in the contralateral joint. By contrast, MRL/MpJ mice were almost completely protected from articular cartilage degeneration in this model. Consistent with previous reports [18], STR/ort showed higher trabecular bone volume fraction (BV/TV) compared to the other two strains. However, previous studies suggested that cartilage degeneration is independent of the underlying bone mass [15]—a hypothesis with diverging opinions in the field.

It has been suggested that the inflammatory response resulting from joint injury may be a significant factor in the progression of PTOA [19]. Studies have shown that STR/ort mice, a model for spontaneous osteoarthritis, exhibit elevated levels of both local and systemic inflammatory markers. Serum analysis showed elevated expression of several cytokines, including Il1β, Ccl4, and Il5, in STR/ort mice compared with that of CBA mice [20]. There is also evidence that MRL/MpJ mice have reduced inflammation, which may play a role in protecting these mice from PTOA [21]. Compared to C57BL/6 mice, MRL/MpJ mice had lower mRNA expression of *Tnfα* and *Il1b* in the synovial tissue and lower protein levels of Il1a and Il1b in the synovial fluid, serum, and joint tissues [21]. Consistent with these observations, our RNA-seq analysis showed that uninjured STR/ort joints expressed elevated inflammatory markers, including *Il1b*, *Il6*, *Ccl2*, and *Cxcl1* and MRL/MpJ had the lowest expression values for these genes (Figure 2C). Joint injury further amplified the expression of inflammatory-response-related genes at 1-day post-injury, which was greater in STR/ort than in the other two strains, and this inflammatory response persisted, while many of these genes reversed to pre-injury levels in C57BL/6J and MRL/MpJ shortly thereafter (Table 1, Figure 4A). This elevated, persistent inflammation may contribute significantly to the enhanced PTOA phenotype observed in STR/ort.

We observed that a number of genes associated with “T cell activation” were highly expressed in MRL/MpJ super-healer mice. However, immunodeficient MRL.RAG1 knockout mice were able to show complete ear hole closure, indicating that the regenerative response is not dependent on T or B cells in the ear [22]; it remains to be determined whether the same holds true for injured joints. We also observed higher expression of mast cell protease *Tpsab1* in both injured and uninjured joints of MRL/MpJ compared to the other two strains at all timepoints (Figure S2), which correlates elevated mast cells with the enhanced healing observed in this mouse strain. Another gene highly expressed in MRL/MpJ compared to the other two strains—*B4galnt2*—has been shown to play a role in skeletal muscle growth in response to acute muscle injury [17] (Figure 6B). We hypothesize that *B4galnt2* also contributes to enhanced healing in MRL/MpJ, and future studies will address the role of this gene in PTOA. The regenerative healing characteristics of the MRL/MpJ strain can also be attributed to reduced expression of apoptosis-associated genes. STR/ort had the highest expression of several apoptosis-associated genes, including *Fos*, *Jun*, and *Id3*, whereas MRL/MpJ had the lowest expression (Figure S1A).

In the mammalian adult, default response to injury involves inflammation, replacement of mature cells, and the formation of scar tissue. Healing in the MRL/MpJ appears more fetal-like with the formation of a blastema, healing without scarring, and with the replacement of lost tissue by functionally and architecturally normal tissue [23]. Remodeling and degradation of the ECM by MMPs is a key step in wound healing. Gourevitch et al. have shown that Mmp2 and Mmp9 protein levels were upregulated in the MRL/MpJ healing ear hole tissue compared with the C57BL/6 and that the MMP activity correlated with blastema formation in the regenerating ear holes [23]. It has also been shown that MRL/MpJ mice exhibit elevated levels of Mmp2, -9, and -14 in the retina compared to C57BL/6 and this elevated MMP expression creates a permissive environment for retinal regeneration in MRL/MpJ mouse [24]. Contrary to these prior findings, we determined that mRNA levels of *Mmp2*, *Mmp3*, and *Mmp14* were significantly lower in the knee joints of MRL/MpJ compared to C57BL/6J and STR/ort joints (Figure 5, Figure S3). Although the expression of these catabolic enzymes elevated in response to injury in all three strains, the expression of these genes remained significantly lower in the injured MRL/MpJ joints compared to injured C57BL/6J and STR/ort joints. These results may point out fundamental differences due to anatomical location and function. MMPs are critical for cartilage remodeling in joints, and elevated levels of these molecules in the joint are usually correlative of enhanced cartilage catabolic activity or degradation in OA [25,26]. Flannelly et al. have shown that mRNA levels of *Mmp2*, *Mmp3*, *Mmp7*, *Mmp9*, *Mmp13*, and *Mmp14* were higher in STR/ort than in age-matched CBA mice at various ages [27]. Consistent with their findings, we found higher levels of MMPs and other matrix degrading enzymes, such as *Adamts1*, *Adamts3*, and *Adamts4*, in injured STR/ort relative to the other two injured strains (Figure 5), suggesting that elevated levels of these matrix degrading enzymes may contribute to enhanced cartilage degradation in STR/ort, and lower levels in MRL/MpJ may be one mechanism by which this strain is resistant to PTOA.

*Ankrd1*, a transcriptional repressor of *MMP13* [28], was highly upregulated in MRL/MpJ at 1-day post-injury, whereas only moderately changed in C57BL/6J and STR/ort (Figure 4B). Global deletion of *Ankrd1* resulted in delayed excisional wound closure [29]. Deletion of *Ankrd1* also resulted in moderate downregulation of *Mmp2* and *Mmp14* [28]. Ankrd1 also plays an anti-inflammatory role through feedback inhibition of NF-κB transcriptional activity [30]. These findings suggest that *Ankrd1* may play a role in protecting MRL/MpJ against injury-induced cartilage damage, possibly by keeping the MMP expression at a low level. MAM Domain Containing 2 (*Mamdc2*), a gene encoding an ECM protein, had extremely low expression in MRL/MpJ but was moderately expressed in the other two strains (Figure S2). *Mamdc2* was upregulated in injured STR/ort joints compared to uninjured contralateral joints at 2-weeks post-injury. Interestingly, we also found *Mamdc2* to be upregulated in C57BL/6J at 6- and 12-weeks post-injury [10]. Furthermore, *MAMDC2* was significantly upregulated in human OA samples [31], which positions MAMDC2 as an ideal candidate biomarker for PTOA. Peroxidasin (*Pxdn*) is another potential OA biomarker identified in this study that was also upregulated in human OA [31]. Very little is known about the functions of these genes, and they warrant further investigation.

One limitation of our study is that we sequenced the whole joints instead of individual tissues of the joint, which makes it difficult to tease out the cellular source of the gene expressions observed. These challenges may be overcome by examining candidate proteins for their tissue-specific expression using other techniques such as immunohistochemistry. Another limitation is that we used the contralateral joint as controls instead of age-matched sham injured joints; this may have caused us to underestimate changes mediated by the injury that had systemic effects on both joints. Regardless, this study identified hundreds of genes and several new pathways that may contribute to PTOA pathogenesis and should be further evaluated in forthcoming studies. Our study provides novel insights into genes and molecular pathways involved in the early stages of PTOA development and identifies several putative candidate genes that may contribute to enhanced healing observed in MRL/MpJ. In addition, the data generated in this study could help facilitate future research in the identification and development of novel approaches to treat PTOA.

## 4. Materials and Methods

### 4.1. Animals and Tibial Compression (TC) Joint Injury

Right knee joints of 10-weeks-old STR/ort, C57BL/6J (Jackson Laboratory, Bar Harbor, ME, USA; Stock No: 000664), and MRL/MpJ (Jackson Laboratory, Bar Harbor, ME, USA; Stock No: 000486) mice were injured using a compressive load of 10–12N, as previously described [9,10]. Mice were anesthetized via isoflurane inhalation and placed in a prone position with right tibias vertically aligned between two platens for tibial compression. ACL rupture was produced via a single dynamic axial compressive load at 1 mm/s using an electromagnetic material testing machine (ElectroForce 3200, TA Instruments, New Castle, DE, USA). Buprenorphine analgesia was administered immediately post-injury (0.01 mg/kg). All animal experimental procedures were completed in accordance with the Institutional Animal Care and Use Committee (IACUC) guidance at Lawrence Livermore National Laboratory under approved protocol 168 (last approval date 5/21/2018).

### 4.2. Histological Assessment of Articular Cartilage and Joint Degeneration

Injured and uninjured (contralateral) joints were collected at 6- and 12-weeks (*n* > 5 per group) post-injury. Joints were dissected, fixed in 4% paraformaldehyde, decalcified using 0.5 M EDTA, infiltrated in increasing concentrations of isopropanol, equilibrated into mineral oil, and embedded into paraffin wax. Six-micrometer paraffin sections were stained on glass slides using 0.1% Safranin-O and 0.05% Fast Green using standard procedures (IHC World, Woodstock, MD, USA) and imaged using a Leica DM5000 microscope. Three blind reviewers independently assessed OA severity using a modified OARSI [scale to examine the medial compartment of injured and uninjured joints (sagittal views); grade scale 0–0.5 normal; 1–2 mild; 3–4 moderate; 5–6 severe cartilage damage]. All sagittal sections were collected on the medial compartment of the knee. For uninjured (contralateral) samples, the evaluation was performed by examining 6 µm serial sections between 400–600 µm in depth from the joint surface. For injured joints, due to the osteophyte and dislocation, histological evaluations were carried out on serial sections between 700–900 µm in depth. However, because MRL injured joints had reduced ectopic bone and joint dislocation, these samples were evaluated between 400–600 µm in depth to obtain comparable regions in the joint.

### 4.3. Microcomputed Tomography Analysis and Osteophyte Quantification

Whole knee joints (*n* > 5 per group) were scanned using μCT (SCANCO μCT 35, Brüttisellen, Switzerland) according to rodent bone structure analysis guidelines (X-ray tube potential = 55 kVp, intensity = 114 μA, 10 μm isotropic nominal voxel size, integration time = 900 ms) [32]. Trabecular bone in the distal femoral epiphysis was analyzed by manually drawing contours on 2D transverse slices. The distal femoral epiphysis was designated as the region of trabecular bone enclosed by the growth plate and subchondral cortical bone plate. Osteophyte volume in joints was quantified by drawing contours around all heterotopic mineralized tissue attached to the distal femur and proximal tibia as well as the entire patella, fabellae, and menisci; the patella, fabellae, and menisci of contralateral limbs were also contoured. Total mineralized osteophyte volume was then determined as the volumetric difference in mineralized tissue between injured and uninjured joints. Statistical analysis was performed using a paired *t*-test to compare injured and contralateral knees.

### 4.4. RNA Sequencing and Data Analysis

Injured and contralateral joints (*n* > 4 per group) were dissected and cut at the base edges of femoral and tibial joint regions with small traces of soft tissues to preserve the intact knee joint. The RNA was isolated and sequenced as previously described [10]. RNA-seq data quality was checked using FastQC (version 0.11.5) software. Sequence reads were aligned to the mouse reference genome (mm10) using TopHat (version 2.0.11) [33,34]. After read mapping, “featureCounts” from Rsubread package (version 1.22.2) [35] was used to perform summarization of reads mapped to RefSeq genes, and gene-wise read counts were generated. Genes were filtered from downstream analysis if they did not have read count of at least 2 in at least five libraries. RUVseq [36] was used to normalize data using 25 housekeeping genes (Supplementary Table S4). Differentially expressed genes were identified using edgeR (version 3.14.0) [37]. A gene was considered significantly differentially expressed when its false discovery rate (FDR) corrected *p*-value was less than 0.05 and fold change was greater than 1.5. Heatmaps were generated using heatmap.2 function in R package ‘gplots’. Human OA RNA-seq data was obtained from Steinberg et al. [31].

### 4.5. Immunohistochemistry

Sagittal serial sections were stained utilizing primary antibodies against B4galnt2 (Novus Biologicals, Littleton, CO, USA). Trypsin/EDTA was used for antigen retrieval for 25 min at 37 °C. Antibody staining was performed as previously described [38]. Negative control slides were incubated with secondary antibody only. Stained slides were mounted with Prolong Gold with DAPI (Molecular Probes, Eugene, OR, USA). ImagePro Plus V7.0 Software and a QIClick CCD camera (QImaging, Surrey, BC, Canada) were used for imaging and photo editing.

### 4.6. Functional Annotation

Gene ontology analysis was performed using ToppGene [39] and enriched gene ontology terms and pathways (*p*-value < 0.01) were identified. Cytoscape was used for pathway visualization [40].

## 5. Conclusions

Our data suggest that prolonged inflammation and enhanced expression of matrix degrading enzymes may contribute to a severe PTOA phenotype. This study identified many new potential therapeutic targets, including *B4galnt2*, and potential OA biomarkers, including *Mamdc2* and *Pxdn*. This study also highlighted several candidate genes that may contribute to enhanced healing and/or tissue regeneration.

## Figures and Tables

**Table 1:** Cytokines upregulated in knee joints after anterior cruciate ligament (ACL) injury. Fold upregulation (log2 scale) in injured joints compared to uninjured joints are shown in the table. ns: not significantly differentially expressed.

	MRL			BL6			STR		
*Gene*	1D	1W	2W	1D	1W	2W	1D	1W	2W
*Ccl2*	3.61	ns	ns	3.23	ns	ns	3.53	1.26	1.14
*Ccl7*	4.52	2.27	ns	3.73	1.97	ns	4.21	1.58	2.02
*Ccl8*	2.52	2.9	ns	1.66	1.81	ns	1.04	1.23	1.84
*Ccl17*	ns	ns	ns	ns	ns	ns	ns	ns	1.99
*Ccl19*	ns	ns	ns	0.67	ns	ns	ns	ns	ns
*Ccl20*	ns	ns	ns	3.24	ns	ns	4.73	4.27	ns
*Ccl28*	ns	ns	ns	0.72	ns	ns	ns	ns	ns
*Csf1*	0.69	ns	ns	ns	ns	ns	0.59	ns	ns
*Cxcl1*	2.8	ns	ns	2.59	ns	ns	3.02	1.51	1.87
*Cxcl2*	ns	ns	ns	ns	ns	ns	2.86	2.13	ns
*Cxcl3*	ns	ns	ns	3.23	ns	ns	4.17	1.81	ns
*Cxcl5*	1.53	ns	ns	2.48	ns	ns	3.06	1.18	ns
*Cxcl14*	1.52	0.59	ns	0.97	ns	ns	1.87	ns	1.18
*Cxcl16*	1.06	0.91	ns	1.72	0.88	ns	0.83	1.17	0.78
*Il1b*	ns	ns	ns	ns	ns	ns	0.64	ns	ns
*Il5*	ns	ns	ns	ns	2.03	ns	ns	ns	ns
*Il6*	2.29	ns	ns	2.29	ns	ns	3.24	1.73	1.95
*Il11*	2.7	1.68	ns	2.82	1.67	2.02	4.01	2.28	2.82
*Il17d*	ns	1.15	ns	ns	1.01	ns	ns	ns	ns
*Il33*	1.17	1.5	ns	1.71	1.06	1.2	1.28	1.58	1.25
*Lif*	1.16	ns	ns	ns	ns	ns	1.16	ns	ns
*Tnf*	ns	ns	ns	ns	ns	ns	0.77	ns	ns
*Tnfsf9*	ns	ns	ns	1.27	ns	ns	ns	ns	ns
*Tnfsf15*	ns	ns	ns	ns	1.52	ns	ns	1.04	ns
*Tnfsf18*	ns	ns	ns	ns	ns	ns	1.58	2.26	1.52

**Table 2:** Metallopeptidases upregulated in knee joints after ACL injury. Fold upregulation (log2 scale) in injured joints compared to uninjured joints are shown in the table. ns: not significantly differentially expressed.

	MRL			BL6			STR		
*Gene*	1D	1W	2W	1D	1W	2W	1D	1W	2W
*Adam5*	ns	ns	ns	ns	ns	ns	ns	0.78	ns
*Adam9*	ns	ns	ns	0.96	ns	ns	ns	0.66	ns
*Adam12*	0.65	0.73	ns	0.96	1.11	ns	0.72	1.6	0.73
*Adam23*	ns	1.1	0.96	1.01	1.04	1.13	ns	1.55	1.66
*Adamts1*	0.71	ns	ns	1.02	ns	ns	1.31	1.01	ns
*Adamts3*	ns	ns	ns	ns	0.83	ns	ns	0.99	0.73
*Adamts4*	1.81	1.21	ns	1.66	1.56	ns	1.64	1.73	1.43
*Adamts6*	ns	0.67	ns	ns	0.97	ns	ns	0.63	0.77
*Adamts7*	ns	ns	ns	ns	0.71	ns	ns	0.66	0.94
*Adamts8*	1.27	ns	ns	ns	ns	ns	ns	ns	ns
*Adamts12*	ns	1.47	ns	1.02	1.61	0.96	ns	1.6	1.11
*Adamts14*	ns	ns	ns	ns	0.64	ns	ns	0.71	ns
*Adamts15*	ns	1.31	0.84	ns	0.97	0.79	ns	0.64	1.35
*Adamts16*	ns	3.05	2.6	1.08	3.75	3.37	ns	1.52	3.35
*Adamts17*	ns	ns	ns	ns	ns	0.7	ns	ns	ns
*Adamtsl1*	ns	0.84	ns	0.86	0.99	0.62	0.62	1.33	0.98
*Adamtsl2*	1.48	ns	ns	ns	ns	ns	0.86	ns	ns
*Adamtsl3*	0.87	1.11	ns	1.37	0.95	ns	ns	1.25	0.59
*Adamtsl4*	ns	1.01	ns	0.61	0.68	ns	ns	ns	ns
*Aebp1*	ns	1.54	0.88	1	1.41	0.84	ns	1.54	1.16
*Agbl2*	ns	1.38	ns	1.41	1.73	ns	ns	1.69	ns
*Anpep*	ns	1.44	0.75	1.32	1.58	1.08	ns	1.18	1.44
*Cpxm1*	ns	1.27	0.77	ns	1.47	0.85	ns	1.35	0.82
*Cpxm2*	ns	1.39	0.91	ns	1.31	1.1	ns	1.43	1.36
*Dpep2*	1.51	0.83	ns	1.61	0.7	ns	1.03	0.62	0.95
*Mmp2*	ns	1.36	1.02	ns	1.67	1.15	ns	1.31	1.42
*Mmp3*	2.39	2.88	2.42	1.99	2.36	2.12	1.97	ns	2.57
*Mmp11*	ns	ns	ns	ns	ns	ns	ns	0.83	ns
*Mmp12*	ns	ns	1.39	1.62	1.26	1.51	ns	1.44	1.24
*Mmp14*	ns	0.91	ns	ns	1.26	0.66	ns	1.51	1.08
*Mmp19*	1.22	1.42	0.73	1.4	1.12	1.01	0.98	0.92	1.69
*Naalad2*	ns	ns	ns	ns	ns	ns	ns	0.66	ns
*Pappa2*	0.8	ns	ns	ns	0.66	0.83	ns	ns	0.85
*Tll1*	ns	0.87	ns	0.95	1.12	1.01	0.87	1.41	0.96
*Trabd2b*	ns	0.61	ns	0.64	0.97	1.04	ns	ns	0.81

**Table 3:** Genes differentially expressed in MRL/MpJ compared to both C57BL/6J and STR/ort in knee joints and compared to C57BL/6J in digits.

Gene Symbol	Gene Name
Genes with higher expression in MRL/MpJ compared to C57BL/6J and STR/ort	
*Tpsab1*	tryptase alpha/beta 1
*Ccdc38*	coiled-coil domain containing 38
*Aox4*	Aldehyde oxidase 4
*B4galnt2*	Beta-1,4-*N*-acetyl-galactosaminyl transferase 2
*Vwa5a*	von Willebrand factor A domain containing 5A
Genes with lower expression in MRL/MpJ compared to C57BL/6J and STR/ort	
*Trim12a*	tripartite motif-containing 12A
*Mamdc2*	MAM domain containing 2
*Serpina3b*	serine (or cysteine) peptidase inhibitor, clade A, member 3B
*Rab6b*	RAB6B, member RAS oncogene family
*Capg*	capping protein (actin filament), gelsolin-like
*Myoc*	Myocilin
*Fam171b*	family with sequence similarity 171, member B
*H2-D1*	histocompatibility 2, D region locus 1
*Slc15a2*	solute carrier family 15 (H+/peptide transporter), member 2
*Ccdc109b*	Coiled-coil domain containing 109B
*Thnsl2*	Threonine synthase-like 2
*Pccb*	propionyl Coenzyme A carboxylase, beta polypeptide
*Gpx3*	glutathione peroxidase 3
*Ezh1*	enhancer of zeste 1 polycomb repressive complex 2 subunit
*Acsf2*	Acyl-CoA synthetase family member 2
*Pycard*	PYD and CARD domain containing

## Supplementary Materials

Supplementary materials can be found at http://www.mdpi.com/1422-0067/19/9/2657/s1.

## Abbreviations

ACL	Anterior cruciate ligament
ECM	Extracellular matrix
MMP	Metalloproteinase
OA	Osteoarthritis
PTOA	Post-traumatic osteoarthritis
RNA-seq	RNA sequencing
μCT	Microcomputed tomography
