# Supplementary material for: Comparative Transcriptomics Identifies Novel Genes and Pathways Involved in Post-Traumatic Osteoarthritis Development and Progression
Source: Int J Mol Sci. 2018 Sep 7;19(9):2657. doi: 10.3390/ijms19092657 (PMC6163882; doi:10.3390/ijms19092657)
Supplement: Supplementary file 1 [file ijms-19-02657-s001.zip › Figure S1.docx]

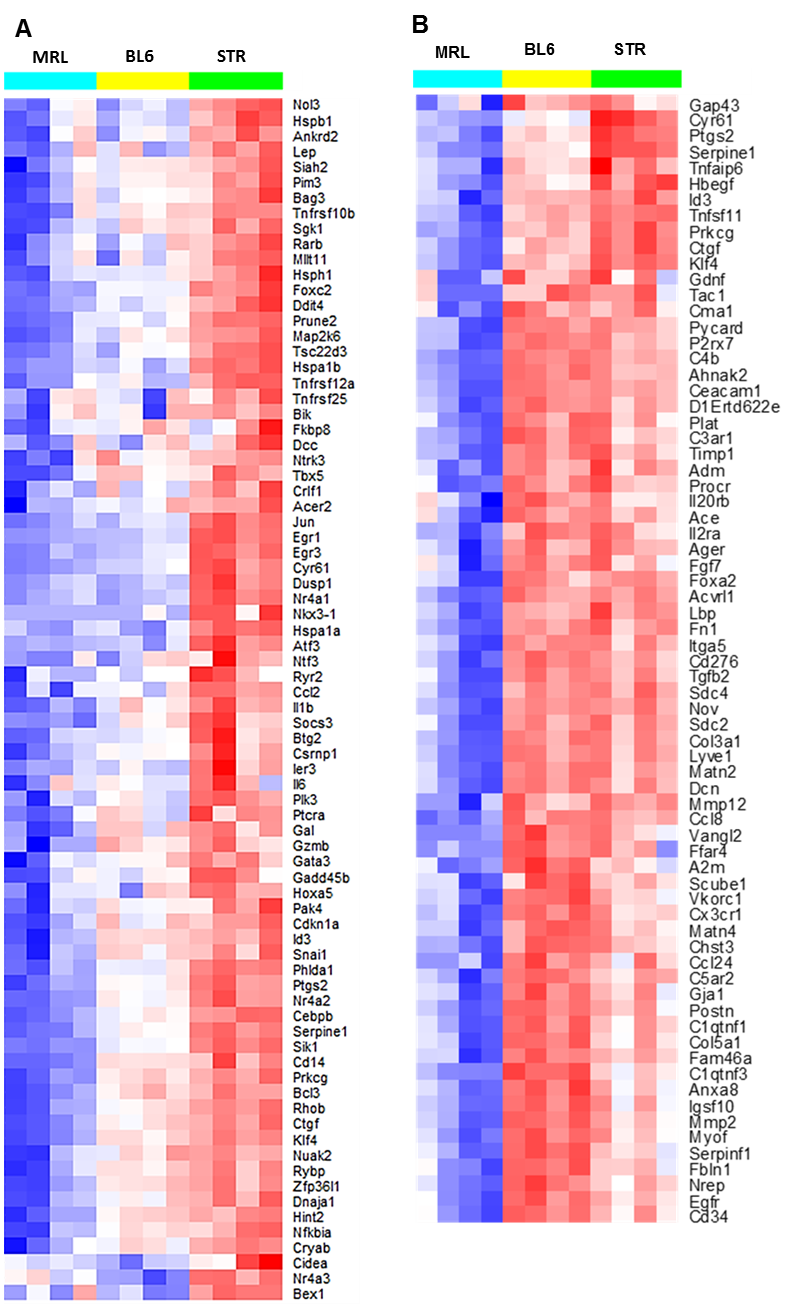
 Figure S1: A) Apoptosis associated genes up-regulated in STR/ort compared to the other two strains. B) Genes associated with wound healing with lowest expression in MRL/MpJ.
