# Supplementary material for: Comparative Transcriptomics Identifies Novel Genes and Pathways Involved in Post-Traumatic Osteoarthritis Development and Progression
Source: Int J Mol Sci. 2018 Sep 7;19(9):2657. doi: 10.3390/ijms19092657 (PMC6163882; doi:10.3390/ijms19092657)
Supplement: Supplementary file 1 [file ijms-19-02657-s001.zip › Figure S2.docx]

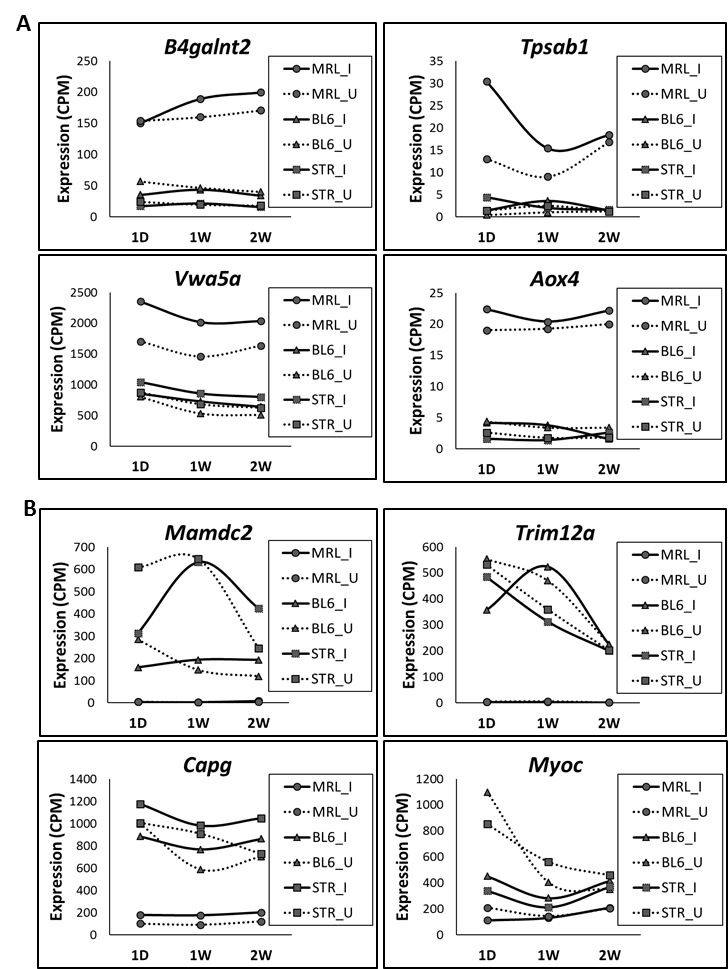


Figure S2: Putative candidates associated with enhanced healing and/or cartilage regeneration in MRL/MpJ. A) Genes highly expressed in MRL/MpJ compared to STR/ort and C57BL/6. B) Genes with lowest expression in MRL/MpJ compared to STR/ort and C57BL/6.
