# Supplementary material for: Comparative Transcriptomics Identifies Novel Genes and Pathways Involved in Post-Traumatic Osteoarthritis Development and Progression
Source: Int J Mol Sci. 2018 Sep 7;19(9):2657. doi: 10.3390/ijms19092657 (PMC6163882; doi:10.3390/ijms19092657)
Supplement: Supplementary file 1 [file ijms-19-02657-s001.zip › Table S4.docx]

**Table S4**: Housekeeping genes used for RNA-seq data normalization.

| **Gene ID** | **Gene Symbol** |
| --- | --- |
| 15452 | *Hprt* |
| 26961 | *Rpl8* |
| 19951 | *Rpl32* |
| 100503670 | *Rpl5* |
| 20016 | *Polr1c* |
| 20018 | *Polr1d* |
| 68052 | *Rps13* |
| 72508 | *Rps6kb1* |
| 15516 | *Hsp90ab1* |
| 27367 | *Rpl3* |
| 76808 | *Rpl18a* |
| 14433 | *Gapdh* |
| 12010 | *B2m* |
| 67891 | *Rpl4* |
| 11461 | *Actb* |
| 18245 | *Oaz1* |
| 19988 | *Rpl6* |
| 19989 | *Rpl7* |
| 11837 | *Rplp0* |
| 20084 | *Rps18* |
| 110651 | *Rps6ka3* |
| 54127 | *Rps28* |
| 66480 | *Rpl15* |
| 19941 | *Rpl26* |
| 66489 | *Rpl35* |
