# Supplementary figures and images for: Comparative Transcriptomics Identifies Novel Genes and Pathways Involved in Post-Traumatic Osteoarthritis Development and Progression
Source: Int J Mol Sci. 2018 Sep 7;19(9):2657. doi: 10.3390/ijms19092657 (PMC6163882; doi:10.3390/ijms19092657)

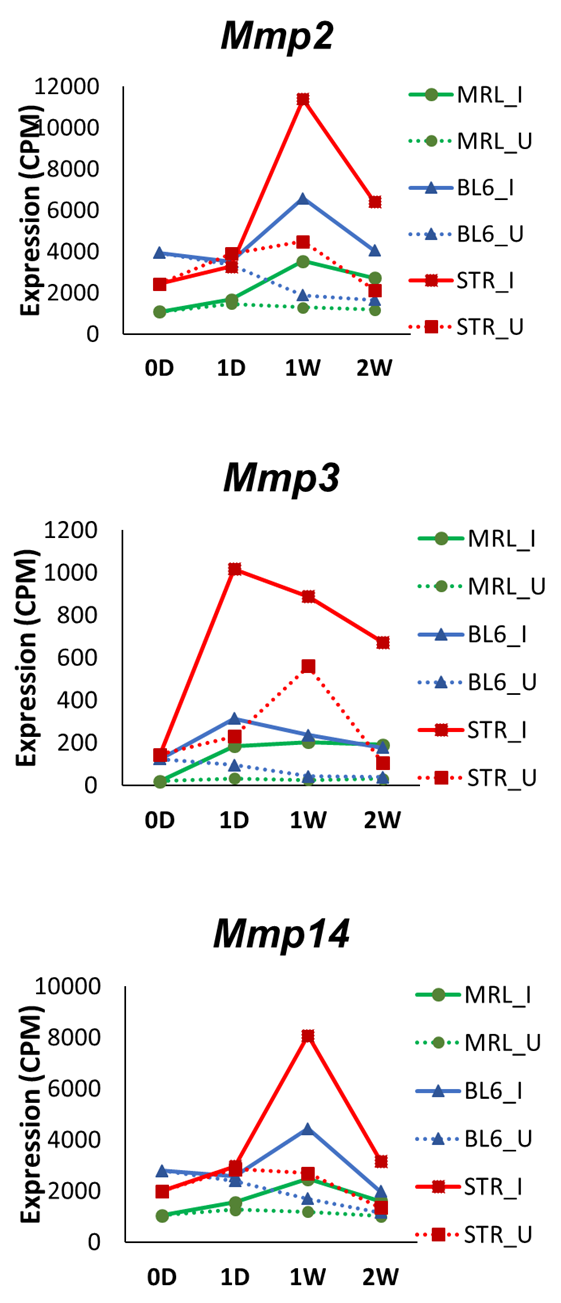


Figure S3: Expression profiles of MMPs. *Mmp2*, *Mmp3* and *Mmp14* showed highest expression in STR/ort.

Supplement: Supplementary file 1 [file ijms-19-02657-s001.zip › Figure S3.docx]
